# Immunosuppression: Cause for Failures of Vaccines against African Trypanosomiases

**DOI:** 10.1371/journal.pntd.0002090

**Published:** 2013-03-14

**Authors:** Henry Tabel, Guojian Wei, Harold J. Bull

**Affiliations:** 1 Department of Veterinary Microbiology, University of Saskatchewan, Saskatoon, Saskatchewan, Canada; 2 Department of Microbiology and Immunology, University of Saskatchewan, Saskatoon, Saskatchewan, Canada; New York University School of Medicine, United States of America

Antibodies to the variant surface glycoprotein (VSG) are required for the control of African trypanosomes infecting the blood, whereas infections of the skin by low numbers of trypanosomes are controlled by innate resistance and do not require antibodies for their control. Low numbers of trypanosomes infecting the skin, although being killed by innate resistance, do not induce protection, but enhance susceptibility to re-infections due to suppression of innate resistance by adaptive immune responses.

We propose to pursue a vaccine strategy that overcomes the induction of immunosuppression but induces a Th1 imprint for protective immunity. We suggest that intradermal immunization with an optimally low dose of antigens of the whole parasite is necessary but not sufficient. We suggest that the immunization has to be accompanied by a treatment that inhibits the arginase pathway of antigen-presenting cells (APCs), but modestly enhances their inducible nitric oxide synthase (iNOS) pathway to induce Th1 memory cells specific for crucial common antigens, which enhance innate resistance.

## Introduction

**Figure 1:**
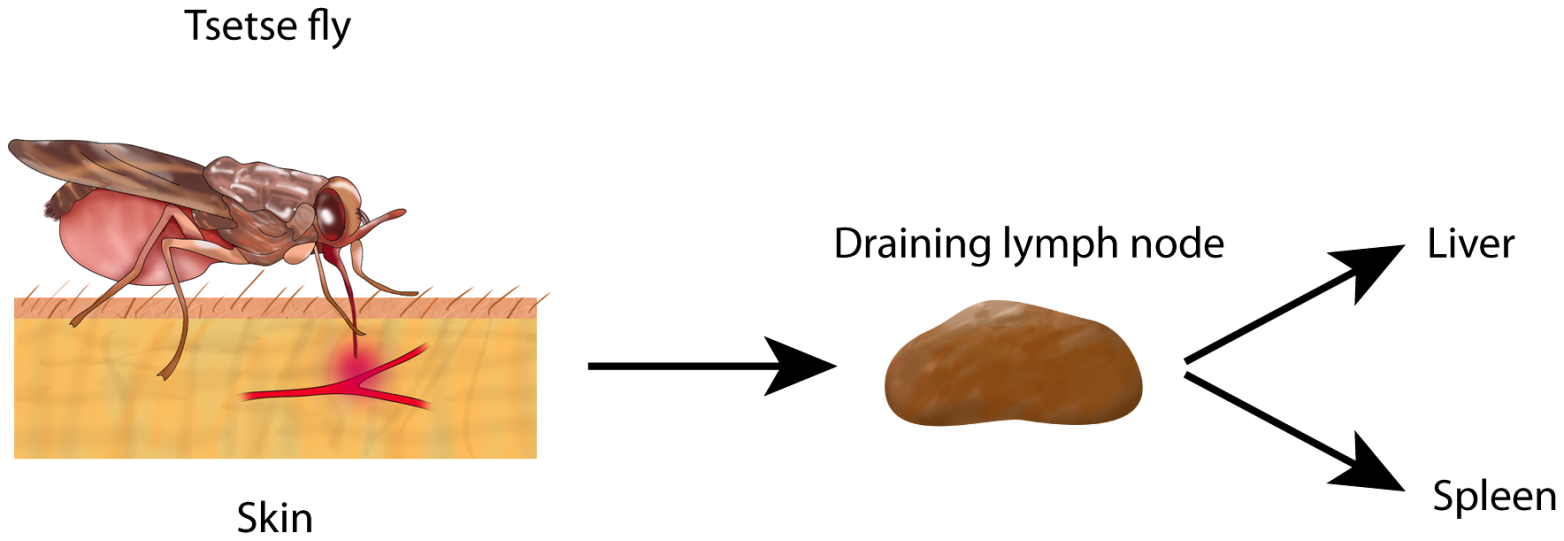
Mode of natural infections by African trypanosomes. Infected tsetse flies bite the host by inserting the proboscis into the skin, inject saliva into the site, and puncture a small blood vessel, resulting in a small hemorrhage. The tsetse fly depicted here is sucking blood from the hemorrhage. During this process, trypanosomes are deposited into the skin. Trypanosomes enter the lymph system and then reach the draining lymph node and the bloodstream. Trypanosomes will circulate in the bloodstream. Whole trypanosomes or fractions thereof end up in macrophages of liver and spleen by antibody- and/or complement-mediated phagocytosis.

African trypanosomes are extracellular hemoprotozoa that cause disease in humans and livestock. *Trypanosoma brucei gambiense* and *T. b. rhodesiense* cause sleeping sickness in humans, also called human African trypanosomiasis (HAT), an emerging disease in East and Central Africa [1], [2]. Infections with *T. congolense*, *T. vivax*, or *T. b. brucei* cause disease in livestock [1]. Various species of tsetse flies (*Glossina* spp.) can harbor African trypanosomes and act as their intermediate hosts. Humans and animals become infected with trypanosomes by bites of infected tsetse flies. A temporary local inflammation, the so-called chancre, develops in the skin at the site of the bite [1]. The trypanosomes move from the skin into the blood via the lymph system (Figure 1).

Mice are susceptible to infections by all African trypanosomes pathogenic for humans or livestock. Thus, infection of mice is a relevant model to study the immunobiology of infections by African trypanosomes.

*Primary intradermal infections by low numbers of parasites* in the skin are controlled by innate resistance mediated by induced nitric oxide (iNO) [3]. At this stage, adaptive immune responses are not protective but are immunosuppressive [3] (discussed below). At the *blood stage of infection*, antibodies are absolutely required for the control of parasitemia [4]–[6]. Antibodies to the VSG control parasitemia by mediating phagocytosis of the trypanosomes by macrophages of the liver and spleen.

## Why Are There No Effective Vaccines?

African trypanosomes have developed a highly sophisticated and complex system of antigenic variation [7]. In the mammalian host, the whole parasite is covered with a coat of about 10^7^ identical molecules of a glycoprotein, the VSG, which is anchored into the cell membrane via a glycolipid, glycosylphosphatidylinositol (GPI) [8], [9]. There is a widely held belief that the almost unlimited capacity for antigenic variation of the surface glycoproteins by the African trypanosomes is the major hurdle for producing a vaccine [5], [10]. In view of our recent experimental results on intradermal infections with low numbers of trypanosomes [3], [11], we do not share this belief.

Past research into the immunobiology of African trypanosomiasis has mostly been based on the immune responses of mice infected intraperitoneally, a route of infection that leads to development of parasitemia [3]–[5], [12]–[14]. Although these studies have provided great insight into the host–parasite relationship, they have neglected to investigate the very early immunological events triggered by the infecting parasites. Thus, we have developed a model for intradermal infections of mice, performed by syringe and needle [3], [11], [15].

Intraperitoneal infections of mice with either *T. brucei* or *T. congolense* lead to infections of the blood and definitely require antibodies to VSG for the control of parasitemia [4]–[6]. Mice are about 100-fold more susceptible to this route of infection than to intradermal infection [3]. Intradermal infections by low numbers (100–500) of African trypanosomes are controlled by innate resistance involving iNO and TNF-α, but require neither antibodies nor T cells for protection [3]. Relevant to these results, it was found that the average man required a minimal dose of 300–450 metacyclic *T. b. rhodesiense* to be infected by the bite of a tsetse fly [16]. Primary intradermal infections are better controlled in CD1d^−/−^ or MHC class II^−/−^ mice, indicating that the innate resistance to low numbers of trypanosomes in primary intradermal infections is suppressed by CD1d-restricted natural killer T cells and MHC class II–restricted T cells, of which the CD1d-restricted natural killer T cells appear to have the most suppressive effect [3].

**Figure 2:**
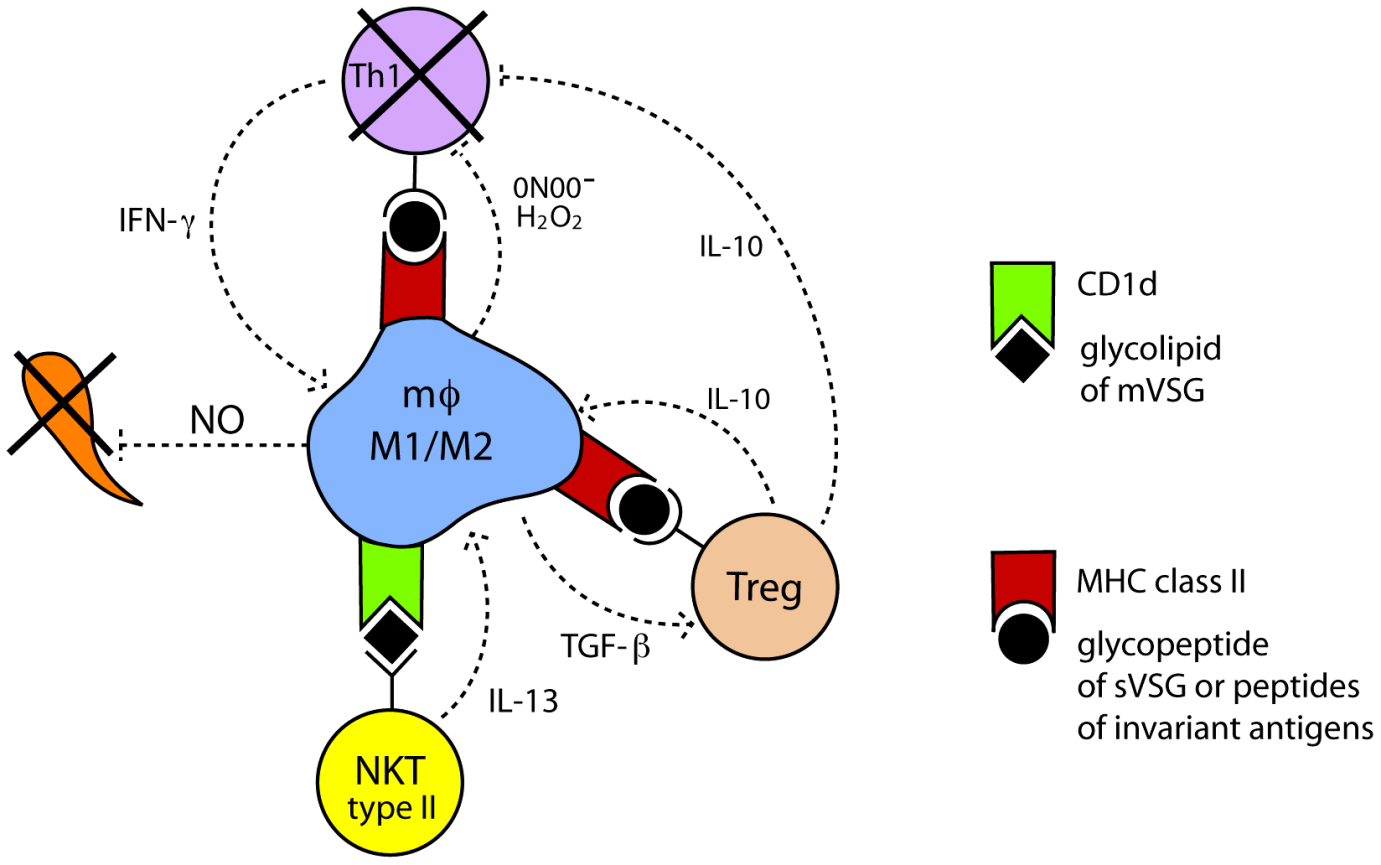
Minimal model: immunosuppression at primary intradermal infections by low numbers of trypanosomes. Macrophages that have engulfed filopodia of trypanosomes [33] or whole killed trypanosomes will process trypanosome antigens and present them at their cell surface. Glycosylphosphatidylinositol (GPI) of membrane variant surface glycoprotein (mVSG) will be presented via CD1d to NKT cells [9], [17]. We argue that the NKT cells are predominantly type II NKT cells that release IL-13 which, in turn, skews the macrophages toward the M2 type. Thus, the antigen-presenting macrophages will predominantly be a mixed M1/M2 type (see text). MHC class II will present peptides to MHC class II–restricted T cells. The microenvironment will skew the naïve MHC class II–restricted T cells towards Tregs [3], [15], presumably via TGF-β produced by macrophages. Tregs, in turn, activate the Arg1 pathway of macrophages by production of IL-10. We propose that many of the naïve trypanosome-specific T cells that develop into Th1 effector cells are deleted by apoptosis, due to peroxynitrite (ONOO-) produced by macrophages under conditions of shortage of L-arginine supply [25], or are functionally impaired by down-regulation of CD3zeta [34].

CD1d is an MHC class I–like molecule that presents glycolipid antigens, such as trypanosomal GPI, to a subset of T cells called natural killer T cells (NKT cells) [9], [17]. There are two subpopulations of NKT cells that vary in the programming of the T cell receptor (TCR): invariant NKT cells (iNKT), type I, and variant NKT cells, type II. Both types of NKT cells recognize, with their TCR, lipids presented by CD1d expressed on the surface of APCs [18]. Type I NKT cells, upon interacting with APCs, predominantly produce IFN-γ and activate the iNOS pathway in the APCs, whereas type II NKT cells produce IL-13 and activate the arginase 1 (Arg1) pathway in APCs [18]. We suspect that the type II NKT cells are predominantly mediating the immunosuppression at intradermal trypanosomal infections (Figure 2).

Primary intradermal infections by 100–500 African trypanosomes that, in fact, are killed by innate resistance, not only fail to generate a long-term protective immunological state, but result in enhanced susceptibility to intradermal challenges [3]. Trypanosome-specific cells of draining lymph node and spleen are primed as early as 24 h after intradermal infection [3]. Surprisingly, intradermal injection of mice with a lysate (trypanosomes killed by sonication) of 10^2^ *T. brucei*, strain Whatat 1.1, does not provide protection but makes such mice more susceptible to an intradermal challenge with 10^2^ *T. brucei* strain 10–26 [3]. The enhanced susceptibility is unrelated to antigenic variation. We also found enhanced susceptibility in mice immunized into the back skin with a cloned and purified peptide of a *T. congolense* protein and challenged by infecting the foot pad [11]. Effector and memory lymphocytes preferentially home to non-lymphoid tissues such as skin [19], [20]. We suggest that intradermal infections with low numbers of trypanosomes or injections with mechanically killed trypanosomes prime the adaptive immune system to suppress protective immunity to an intradermal challenge.

All previous attempts to produce vaccines against African trypanosomes were only partially successful or failed entirely. A comprehensive review on previous vaccination attempts has been published recently [21].

We propose that in any attempt to produce an effective vaccine, it will be crucial to address the problem of induction of immunosuppression by the trypanosomes injected into the skin by infected tsetse flies.

## Immunosuppression in Humans and Animals Infected by African Trypanosomes

### Immunosuppression to Heterologous Antigens

Humans, cattle, and mice infected by African trypanosomes show lower immune responses to vaccines against various bacterial and viral diseases. In mice or cattle infected with *T. brucei* or *T. congolense*, there is reduced proliferation of T cells in response to stimulation by T cell mitogens, such as ConA or PHA, and a reduced antibody response to sheep red blood cells (SRBC) following immunization with SRBC [5], [12], [13], [22].

### Immunosuppression to Trypanosomal Antigens

Sacks and Askonas [6] infected mice with *T. brucei* and tested the anti-VSG antibodies to the different variants after each of three waves of parasitemia. As the infections progressed, IgM and IgG anti-VSG antibody responses declined. IgG antibodies declined more rapidly. After the third parasitemia, only low levels of IgM anti-VSG antibodies were detectable.

## Mechanisms of Immunosuppression

Roelants and Pinder [5] carried out an extensive review and concluded both suppressor macrophages and suppressor T cells are involved in the immunosuppression in mice infected with *T. brucei* or *T. congolense*. Askonas' lab has convincingly shown that macrophages become immunosuppressive after antibody-mediated phagocytosis of *T. brucei* [12].

Nitric oxide (NO) produced by macrophages is a mediator of immunosuppression in *T. brucei* infection of mice [13], [14], [23]. NO is a major mediator of immunosuppression only during the early phase of infection of the blood [13]. It is the stimulation of such macrophages by IFN-γ that, in synergy with TNF-α, induces the synthesis of high amounts of NO [13], [23].

### M1 versus M2 Macrophages

The diverse biological activity of macrophages is mediated by phenotypically distinct subpopulations of cells that develop in response to inflammatory mediators in their microenvironment. Two major populations have been characterized: classically activated M1 macrophages and alternatively activated M2 macrophages [24]. The M1 type develops upon activation by IFN-α/β, IFN-γ, and/or TNF-α. The M2 type develops after activation by IL-10, IL-4, and/or IL-13 [24]. Activation of the inducible nitric oxide synthase (iNOS or NOS2) has been regarded as one of the most specific markers for M1 macrophages and activation of Arg1, the most specific marker of M2 macrophages [24], [25]. Both types of macrophages have been associated with immunosuppression. The L-arginine metabolism in macrophages controls T lymphocyte function [25]. Both the arginase pathway and the iNOS pathway use L-arginine as their substrate. Both pathways compete for the available L-arginine and cross-regulate each other [25]. Despite the distinct expression of iNOS and Arg1 in M1 and M2 macrophages, respectively, some macrophages have been shown to express both iNOS and Arg1 [24]. Thus, macrophages of mixed characteristic do exist.

BALB/c mice are more susceptible to *T. congolense* and *T. brucei* than relatively resistant C57BL/6 mice. In mice intraperitoneally infected with *T. brucei*, arginase mRNA is expressed higher in peritoneal macrophages of infected BALB/c than in those of infected C57BL/6 mice. In co-cultivation with macrophages, *T. brucei* directly induces increased Arg1 and Arg2 mRNA levels in macrophages as well as increases macrophage arginase activity [26]. From 2 days on after infection, arginase activity is increasingly up-regulated in peritoneal macrophages of Swiss mice subcutaneously infected with *T. brucei*. Under the same conditions, increasing iNOS activity is delayed by a couple of days [27].

Immunity to infections is mediated by memory T cells and B cells, which are generated from naïve precursor cells after exposure to the microbial antigens. Upon interaction of naïve T cells with the APC, naïve T cells rapidly proliferate and differentiate into effector T cells. This phase of proliferation lasts about 1 week and is followed by a contraction phase of about 14 days during which about 90% of the effector T cells die, whereas the remaining cells differentiate into memory T cells [28].

Th1 cells mediate resistance to African trypanosomes [22]. In natural infections, the tsetse fly injects the trypanosomes together with fly saliva. Initial injections of tsetse fly saliva induce Th2 responses [29]. The tsetse fly saliva will likely alter the microenvironment of the injection site of the skin, skewing APCs toward activating the Arg1 pathway by IL-4 [24] and thus, like the suppressor T cells, interfere with the innate resistance. We conclude that, in African trypanosomiasis, there is a lack of differentiation of trypanosome-specific Th1 cells into Th1 memory cells specific for variant and common parasite antigens.

We contend that, at the intradermal stage of infection, the immunosuppression is predominantly controlled by a mixed M1/M2 macrophage environment and by suppressor T cells [3], [24], [25] (Figure 2). Although tsetse saliva plays a role in the pathogenesis [29], the effect of saliva has to be bypassed in any vaccine strategy, as has been achieved in the highly successful vaccine against mosquito-transmitted yellow fever [30]. We propose that inhibiting the arginase pathway [27] and adequately supplying L-arginine [25], combined with intradermal immunization with low numbers of trypanosomes, will ameliorate or abolish the immunosuppressive environment, lead to induction of a trypanosome-specific Th1 imprint and, in turn, enhance innate resistance.

Our proposal to use a vaccination procedure that enhances Th1 cell differentiation appears to run counter to the observation that Th1 cell/IFN-γ-induced NO mediates profound immunopathology and immunosuppression in African trypanosomiasis [31]. NO, however, is a double-edged sword. Our reasoning is based on the observation that high concentrations of NO are immunosuppressive, whereas low concentrations of NO enhance Th1 cell differentiation [32].
